# Isolation and Expression Pattern Analysis of *Larix olgensis LoNAC5*: *LoNAC5* Acts as a Positive Regulator of Drought and Salt Tolerance

**DOI:** 10.3390/plants14101527

**Published:** 2025-05-19

**Authors:** Qing Cao, Junjie Du, Mengxu Yin, Chen Wang, Tiantian Zhang, Qingrong Zhao, Lu Liu, Hanguo Zhang, Lei Zhang

**Affiliations:** State Key Laboratory of Tree Genetics and Breeding, Northeast Forestry University, Harbin 150040, China; caoqing1997@nefu.edu.cn (Q.C.); yayuyo@nefu.edu.cn (J.D.); ymx1231@nefu.edu.cn (M.Y.); wangchen0@nefu.edu.cn (C.W.); 20200903@nefu.edu.cn (T.Z.); 18845294870@163.com (Q.Z.); 2067744783@nefu.edu.cn (L.L.)

**Keywords:** *Larix olgensis*, *LoNAC5*, bioinformatics analysis, expression pattern, drought and salt tolerance, molecular mechanism

## Abstract

NAC transcription factors are a kind of plant specific transcription factor widely distributed in plants, and they play an important role in the process of plant growth and development. According to the transcriptome data, a transcription factor with typical NAC characteristics was isolated from *Larix olgensis* (common name “Dahurian larch”), that we named *LoNAC5*. The length of the coding sequence (CDS) was 1164 bp, encoding 387 amino acids. The LoNAC5 protein harbors a NAM (NAC family) domain at the 14–139 aa region of its N-terminus and an activation domain at the 324–364 aa region of the C-terminus. Phylogenetic tree analysis revealed that *LoNAC5* belonged to the ATNAC3 subgroup. Cis-acting element analysis showed that there were multiple plant stress-resistance-related elements on the promoter of *LoNAC5*, including hormone and light responsiveness elements. *LoNAC5* was localized in the nucleus by injection transformation of tobacco leaves. Results suggested that the LoNAC5 protein is active as a homodimer and that it binds to the GATGTG motif. The results of RT-qPCR showed that *LoNAC5* is a highly expressed gene in *L. olgensis*, and the expression level is highest in 180-day needles. *LoNAC5* responded to various hormone treatments and was induced by drought and salt stress. The yeast phenotype test showed that overexpression of *LoNAC5* could make yeast grow better under drought and salt stress. It was speculated that *LoNAC5* might act in *L. olgensis* as a positive regulator of drought and salt tolerance.

## 1. Introduction

The families of the plant-specific transcription factors (TFs) are defined by their characteristic DNA-binding domains [1]. The NAC family of TFs (including NAM, ATAF, and CUC) is a plant-specific transcription factor family, which has a characteristic domain of about 150 amino acids [2,3]. This domain binds specific DNA sequences and is the basis for the classification of NAC family members [4]. Previous studies have found that the conserved domain of NAC protein is generally located in the N-terminus, which can be generally divided into five motifs: A–E sub-domains [5,6,7]. This study found that sub-domains A, C, and D are highly conserved. Among them, C and D contain nuclear localization signals, which is presumed to be related to the nuclear localization in transcription factors and the recognition of specific DNA sequences. In contrast, B and E are varied, and are only conserved in some NAC subgroups, which is presumed to be associated with different functions of NAC TFs [6,7]. The C-terminus of NAC protein usually has simple amino acids with high repeatability (Thr, Ser, Pro, Glu, or other acidic amino acid residues), which makes it have diverse transcriptional activation domains in the C-terminus [8,9].

The NAC transcription factor family is very large. More than 8000 NAC family transcription factors have been found in plants, and 117 and 140 NAC family members have been found in *Arabidopsis thaliana* (L.) Heynh [10] and *Oryza sativa* L. [11] alone. According to the results of sequence clustering analysis, some researchers divided rice NAC family members into three subfamilies: NAM, ATAF, and OsNAC3 [12]. Alternatively, the NAC family was divided into five groups based on phylogenetic analyses in rice: OsNAC7, NAC1, NAM/CUC, GRAB2, and NAC2 [13]. Results of evolutionary studies of NAC family genes in rice and *Arabidopsis* showed that these NAC family proteins were classified into 2 groups and 18 subgroups by sequence similarity [14]. In *Populus* spp. 163 full-length NAC genes were identified that were divided into 18 different subfamilies according to the results of a phylogenetic tree [15]. Gene structure, motif composition, sequence conservation, and function tended to be more similar within than among NAC family subgroups [16,17].

NAC transcription factors have a variety of biological functions, which are not only involved in plant growth and morphogenesis [18,19], but also in response to various stresses [20,21,22]. *NAM* is one of the first discovered NAC family genes, and mutation of this gene in *Petunia* embryos will lead to abnormal development of shoot apical meristem [7]. *CUCs* from *Arabidopsis thaliana* were found to be associated with ovule number [23] and shoot meristem formation [18,24]. *NAM* and *CUC* genes in maize have also been shown to be related to cotyledon formation [25]. *VNDs* have been shown to switch plant metaxylem and protoxylem vessel formation [26,27,28,29]. The overexpression of *AtNAC1* [30] or *GmNAC20* [20] can promote lateral root formation in plants. In addition, researchers have found that NAC family genes can not only regulate plant tolerance to abiotic stresses such as drought [31,32,33], salt [34,35], cold [36,37,38], and high temperature [39], but also participate in plant response processes to biotic stresses such as viruses [40,41,42,43], pathogens [44,45,46], and pests [47,48].

With technological advancements, plant genome sequencing has progressed significantly, leading to a comprehensive analysis of the NAC family genes in most plants [15,49,50]. In *Cryptomeria fortunei* Hooibrenk, 33 *CfNACs* that may be involved in lignin biosynthesis, xylem development, and SCW formation have been identified [51]. In addition, three genes belonging to the NAC family in *Larix olgensis* were also successfully identified and named *LoNAC1–3* [52,53]. However, due to the extensive genome and intricate genetic background of larch, research on NAC genes in this family remains scarce [52]. In this study, the NAC family gene *LoNAC5* and its promoter were identified and isolated from *L. olgensis* based on transcriptome data, and the sequence information was analyzed using bioinformatics methods. The expression characteristics of *LoNAC5* were studied through subcellular localization assay and yeast assay. Furthermore, we examined the expression pattern of *LoNAC5* and characterized its role under drought and salt stress conditions in yeast. This study provides a theoretical and molecular foundation for further exploration of *LoNAC5*’s function.

## 2. Results

### 2.1. LoNAC5 Belongs to the AtNAC3 Subgroup

Using *LoNAC5* amplification primers, DNA fragments of approximately 1000 bp and 3000 bp in length were isolated from the cDNA and gDNA of *L*. *olgensis*, respectively. Sequencing results revealed that the total length of the *LoNAC5* sequence was 3205 bp, consisting of three exons and two introns. The length of *LoNAC5* coding region is 1164 bp, encoding 387 amino acids. The theoretical molecular weight of LoNAC5 protein is 43,740.05 Da, with a predicted theoretical PI of 6.12. The corresponding protein consists of 43 positively charged and 47 negatively charged residues, an aliphatic index of 66.36, an instability index of 49.01, and average hydrophobicity of −0.681. These results indicate that the LoNAC5 protein is unstable and likely to be short-lived.

**Figure 1:**
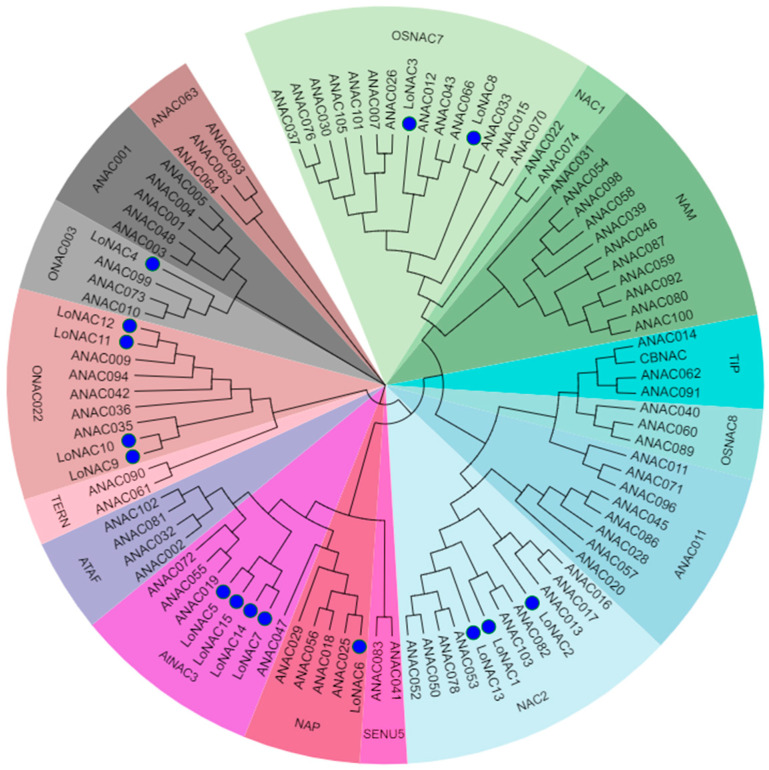
Subgroup classification analysis of *LoNAC5*.

The sequence information of the *L. olgensis* NAC and *Arabidopsis* NAC family protein was obtained from TAIR (The *Arabidopsis* Information Resource) and NCBI (National Center for Biotechnology Information), and the phylogenetic tree was constructed together for homology analysis [14]. The results demonstrated that the 15 *LoNACs* were phylogenetically classified into six distinct subgroups (Figure 1), suggesting functional diversification within the NAC family in larch. Notably, *LoNAC5* clustered within the AtNAC3 subgroup, forming a highly supported clade with *Arabidopsis* stress-responsive NAC members *ANAC019*, *ANAC055*, and *ANAC072*. This evolutionary conservation implies that *LoNAC5* may share functional conservation with its *Arabidopsis* homologs *ANAC019*, *ANAC055*, and *ANAC072*.

**Figure 2:**
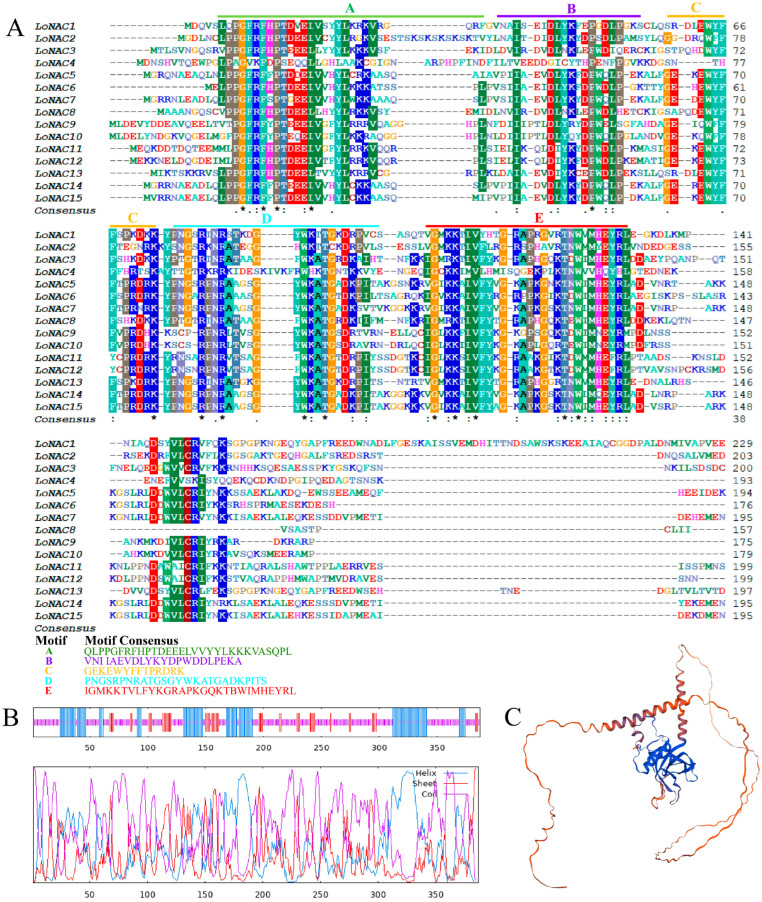
Bioinformatics analysis of LoNAC5 protein. (**A**) The motif analysis of LoNAC5, “*” represents a conserved amino acid; “:” a conservative replacement; “.” a non-conservative replacement; (**B**) secondary structure prediction of LoNAC5; (**C**) tertiary structure prediction of LoNAC5.

Conserved domain (CD)-search analysis identified a conserved NAM domain spanning amino acid residues 14 to 139 in the LoNAC5 protein. Motif analysis showed that the N-terminal domain can be divided into five motifs (Figure 2A), consistent with the structural features of the NAC family. Secondary structure predictions indicated that the encoded protein consisted of 27.13% alpha helices, 13.44% extended chains, and 59.43% random coils (Figure 2B). The SwissMode homology modeling method was used to predict the tertiary structure of LoNAC5 (Figure 2C), and the compared model was a NAC transcription factor (protein: A9NZG8.1, organism: *Picea sitchensis*/*Sitka spruce*/*Pinus sitchensis*) with 94.04% sequence identity. The LoNAC5 protein does not contain signal peptides or transmembrane domains, and its subcellular localization is predicted in the nucleus.

### 2.2. LoNAC5 Conforms to the Characteristics of NAC Transcription Factors

**Figure 3:**
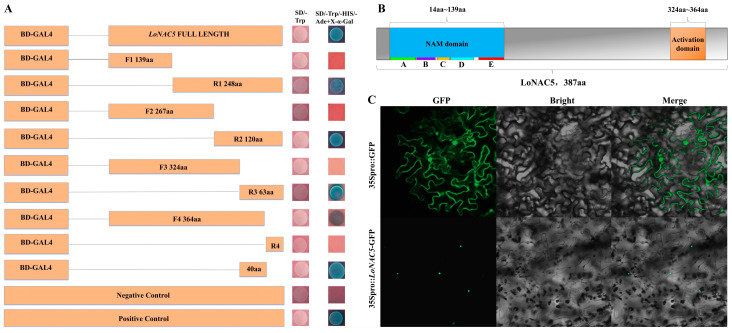
Expression characteristics of LoNAC5 protein. (**A**) Activation domain analysis of LoNAC5; (**B**) structure of LoNAC5 protein; (**C**) the results of subcellular localization observed by laser confocal microscopy.

To determine whether *LoNAC5* has transcriptional activation activity, full-length *LoNAC5* was fused to the GAL4 DNA-binding domain (pGBKT7-*LoNAC5*) and introduced into the Y2HGold yeast strain. The empty vector of pGBKT7 was used as the negative control. The yeast transformed with full-length *LoNAC5* fused to GAL4-BD grew on the selective media without Trp or without Trp, HIS and Ade, indicating that *LoNAC5* exhibits transcriptional activation activity. To further analyze the location of the activation domain, we divided *LoNAC5* into different fragments and inserted them separately into pGBKT7, and then repeated the above operations of transferring into yeast and observing growth status (Figure 3A). The results showed that the activation domain was located at the C-terminus and between F3–R4.

Based on the analysis of conserved and transcriptional activation domains, the structure of the LoNAC5 protein is depicted in Figure 3B. A conserved NAM domain is located between residues 14 and 139 at the N-terminus. This domain can be further divided into five sub-domains. Additionally, a transcriptional activation domain is located between 324 aa and 363 aa at the C-terminus. These findings suggest that *LoNAC5* exhibits the same structural characteristics as most NAC transcription factors described previously.

*LoNAC5* was fused to GFP and was successfully expressed in leaves of *Nicotiana benthamiana*. Observation by laser confocal microscopy revealed that green fluorescence signals could be detected throughout the tobacco epidermal cells in the control group, while in the experimental group (VB191104-*LoNAC5*-GFP), green fluorescence could only be observed in the nucleus of the tobacco epidermal cells (Figure 3C). The results showed that the subcellular localization of *LoNAC5* was in the cell nucleus, which was consistent with the results predicted by WOLF PSORT.

### 2.3. LoNAC5 Can Respond to Drought and Salt Stress

**Figure 4:**
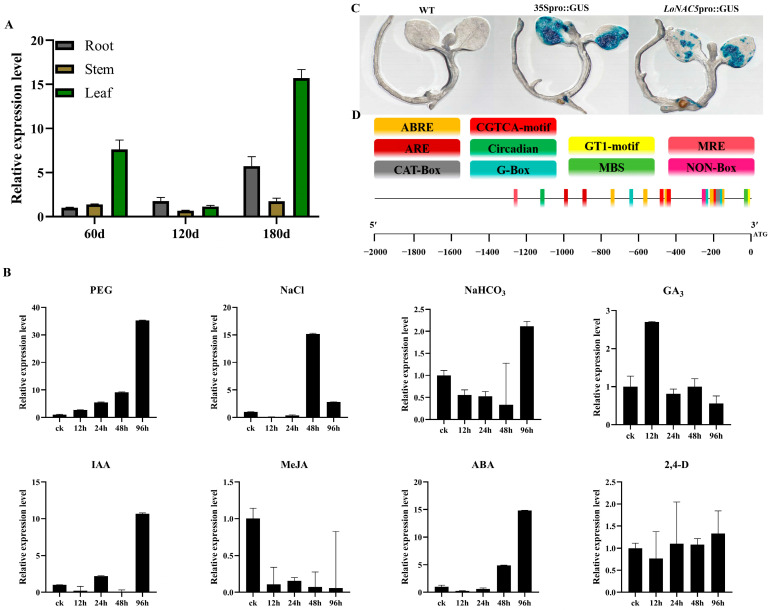
Expression analysis of *LoNAC5* and *LoNAC5* promoter. (**A**) Expression analysis of *LoNAC5* in different growth stages of *L. olgensis*; (**B**) expression analysis of *LoNAC5* under different treatment; (**C**) analysis of promoter activity through GUS staining; (**D**) the cis-acting elements in *LoNAC5* promoter sequence.

The expression pattern of *LoNAC5* in *L. olgensis* was analyzed by reverse transcription quantitative PCR (RT-qPCR). The experiment found that fluorescent signals could be detected in the roots, stems, and leaves of larch at three different stages, indicating that *LoNAC5* was expressed in various parts at each stage. Through the analysis of RT-qPCR results, it can be seen that *LoNAC5* has the highest relative expression level in the leaves of 180-day *L. olgensis* (Figure 4A). The expression level of *LoNAC5* changed under different abiotic stresses. Under the treatment of 28% PEG 6000, the expression level of *LoNAC5* increased with the prolongation of treatment time. The expression level reached the maximum in seedlings after 96 h, which was 35.26 times that of the untreated seedlings. Under the treatment of 0.2 M NaCl solution, the expression level of *LoNAC5* was down-regulated in seedlings treated for 12 h and 24 h, whilst it was up-regulated in seedlings treated for 48 h and 96 h. The relative expression level was the lowest in seedlings treated for 12 h, with a 14.37-fold down-regulation compared to the untreated seedlings. The expression level reached the maximum after 48 h of treatment, with a relative expression level of 15.17 times that of the control. Under the treatment of NaHCO_3_, the change in the expression level of *LoNAC5* was relatively small. The relative expression level of *LoNAC5* was 2.12 times the control at 96 h. *LoNAC5* expression was down-regulated in seedlings treated for 12 h, 24 h, and 48 h. The expression level was the lowest in seedlings treated for 48 h, with a 3-fold down-regulation compared to the untreated seedlings. Under the treatment of 50 mg/L MeJA at four time points, the expression level of *LoNAC5* was down-regulated. The greatest down-regulation occurred in seedlings treated with methyl jasmonate for 96 h, with a 17.28-fold decrease compared to the untreated seedlings. Under the treatment of IAA, the relative expression level was the highest at 96 h, with a 10.66-fold increase compared to the untreated seedlings. At 48 h, the relative expression level was down-regulated by 15.75 times the control. Under the treatment of ABA, the expression level was down-regulated at 12 h and 24 h, and up-regulated at 48 h and 96 h. The relative expression level of *LoNAC5* in seedlings at 96 h was up-regulated by 14.82 times the control. Under the treatments of MeJA, IAA, and ABA, |log2RT| > 3 at multiple time points, indicating more significant changes in the expression level (Figure 4B).

Based on genomic data of *L. kaempferi* (taxid: 54,800), we predicted the sequence information of the *LoNAC5* promoter, which corresponds to the fragment ranging from position 1,076,477 to 1,078,477 of the contig LKA1p01161 (GenBank accession: BSBM01001161.1). The plasmids of the constructed recombinant vector (*LoNAC5*pro::GUS) and empty vector (35Spro::GUS) were expressed separately in tobacco (Figure 4C). GUS stain was not detected in the negative control tobacco (WT). In the experimental tobacco (*LoNAC5*pro::GUS), leaves were a major part of GUS staining, and the intensity and area of GUS staining were lower than those in the positive control tobacco (35Spro::GUS). These results indicate that the *LoNAC5* promoter has a certain initiating activity and is strongest in plant leaves.

The analysis of cis-acting elements revealed the presence of multiple elements with different *LoNAC5* promoter functions as follows: (1) Plant hormone responsive elements: five cis-acting elements are involved in abscisic acid response (ABRE) and methyl jasmonate response (CGTCA motif); (2) Elements involved in plant stress response: two cis-regulatory elements involved in anaerobic induction (ARE) and one MYB binding site involved in drought induction (MBS); (3) Elements related to photosynthesis: four light responsive elements (G-box and GT1 motif) and one MYB binding site participate in light response (MRE). In addition, there are cis-regulatory elements related to circadian rhythm control (Circadian), meristematic expression (CAT box), and meristematic specific activation (NON box) in the *LoNAC5* promoter. Nearly all cis-acting elements are distributed from the starting codon (ATG) to the position 1300 bp upstream, with a significant clustering of these elements around 200 bp and 450 bp upstream of the starting codon (Figure 4D).

### 2.4. Functional Characterization of LoNAC5 in Yeast

**Figure 5:**
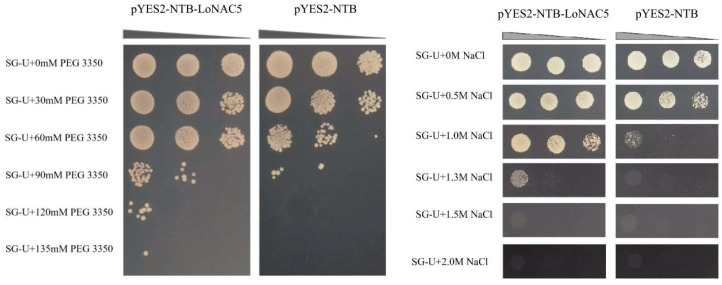
Influence of *LoNAC5* expression in the INVSC1 yeast strain under PEG and salt stress.

The result of RT-qPCR revealed that *LoNAC5* could respond to drought and salt stresses in larch. To validate its role in drought and salt tolerance, *LoNAC5* was functionally characterized in yeast [54]. The negative control yeast (pYES2-NTB) could grow on the media of SG-U + 0 mM PEG 3350, SG-U + 30 mM PEG 3350, SG-U + 60 mM PEG 3350, and SG-U + 90 mM PEG 3350, but could not grow under the conditions of SG-U + 120 mM PEG 3350 and SG-U + 135 mM PEG 3350. However, growth of yeast carrying pYES2-NTB-*LoNAC5* was better than the negative control and could grow under the condition of SG-U + 120 mM PEG 3350. Similarly, the negative control yeast could grow on the culture medium plates of SG-U + 0 M NaCl, SG-U + 0.5 M NaCl, and SG-U + 1.0 M NaCl, but could not grow on the plates of SG-U + 1.3 M NaCl, SG-U + 1.5 M NaCl, and SG-U + 2.0 M NaCl. In contrast, the yeast carrying pYES2-NTB-*LoNAC5* not only could grow on the culture medium of SG-U + 1.3 M NaCl, but also its growth state on the SG-U + 1.0 M NaCl plate was better than that of the negative control (Figure 5). In conclusion, the growth state of the yeast carrying pYES2-NTB-*LoNAC5* was better than the yeast carrying pYES2-NTB, speculating that *LoNAC5* is a positive regulatory factor for salt and drought resistance.

### 2.5. LoNAC5 Can Form Homodimers and Bind to CATGTG Motif

**Figure 6:**
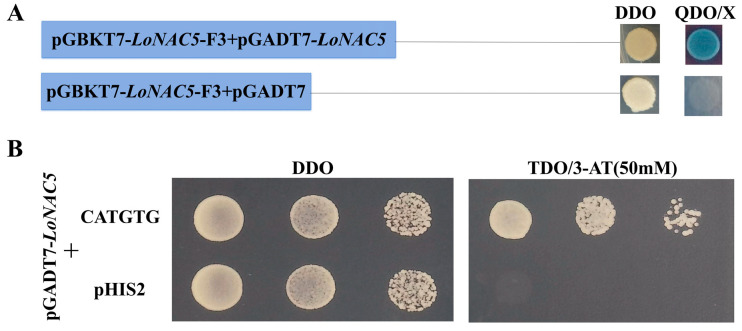
Expression characteristics of *LoNAC5* in yeast. (**A**) Homodimer analysis of *LoNAC5*; (**B**) binding of CATGTG motif to *LoNAC5*.

The yeast carrying PGBKT7-*LoNAC5*-F3 can grow on the QDO (selective media without Trp, Leu, HIS, and Ade) with 40 μL/L X-α-Gal and appears blue when co-transformed with pGADT7-*LoNAC5* (Figure 6A), indicating that *LoNAC5* can form homodimers. NAC transcription factors mainly bind to the NACRS with core sequences or “CATGTG” to regulate gene expression [55]. The NAC proteins *ANAC019/055/072* in the same subgroup as *LoNAC5* bound specifically to the CATGTG motif [6]. The Y187 yeast strain carrying pGADT7-LoNAC5 can grow on the TDO (selective media without Trp, Leu, and HIS) when co-transformed with pHIS2-CATGTG (Figure 6B), indicating that LoNAC5 can bind to the CATGTG motif.

## 3. Discussion

Larch is an important timber tree species in northeast China. However, research on NAC transcription factors in larch remains scarce and warrants further exploration. NAC transcription factors typically exhibit a high degree of conservation at the N-terminus. This conserved domain, usually composed of approximately 150 amino acids, can frequently be partitioned into five distinct sub-domains. In contrast, their C-termini are diverse, and sometimes a transcriptional activation domain can be found therein. The OsNAC19 protein harbors a plant-specific NAC domain a in the N-terminus, and the activation domain has been detected within 181–240 aa of its C-terminal region [56]. NAC transcription factors such as *VND7* [27], *TaNAC8* [57], and *CarNAC5* [58] exhibit highly similar structural characteristics, which are also consistent with the structure of *LoNAC5*: a NAM domain is present from 14 to 139 aa at the N-terminus and a transcriptional activation domain is located from 324 to 364 aa at the C-terminus (Figure 3B). Studies have revealed that NAC transcription factors have conserved intron–exon structure [59], and some NACs in rice and sorghum have been found to have two introns [60,61]. In this experiment, it was found that *LoNAC5* also has two introns, and the coding region is 1164 bp. The LoNAC5-GFP fusion protein was localized in the nucleus, exhibiting typical transcription factor characteristics [58]. Collectively, these results suggest that *LoNAC5*, isolated from the cDNA of *L. olgensis* in this experiment, is a canonical transcription factor of the NAC family.

Drought and salinity, two major forms of abiotic stress, severely disrupt the growth, development, and reproduction processes of plants. As a consequence, this not only undermines the yield and quality of plants but also incurs significant economic losses [53]. NAC transcription factors exhibit a wide array of biological functions. The AtNAC3 subgroup, to which the LoNAC5 protein belongs, has been designated as the SNAC group (stress-responsive NAC) [62]. Previous research has shown that this subgroup is particularly crucial in the plant’s response to abiotic stress [21,62,63]. Through homologous analysis by constructing a phylogenetic tree, it was found that *LoNAC5* and *Arabidopsis ANAC019/055/072* belong to the same subgroup (Figure 1). Overexpressing any one of *ANAC019/055/072* is sufficient to augment the abiotic stress tolerance capabilities of *Arabidopsis thaliana* [64]. Additionally, *PwNAC2*, a NAC transcription factor identified through NCBI sequence alignment and highly homologous to *LoNAC5*, has been shown to enhance the drought and salt tolerance of *Arabidopsis thaliana* when overexpressed [65]. The acquisition of transgenic plants through the in vitro regeneration system of larch is characterized by a protracted timeline. Concurrently, the cell embryogenesis system manifests pronounced instability, accompanied by a notably elevated incidence of somatic embryo malformations [66].

Through bioinformatics analysis, the transcription factor *LoNAC5*, which is related to plant abiotic stress, has been preliminarily identified. The results of RT-qPCR indicated that the expression of *LoNAC5* is induced by drought and salt stress. Remarkably, yeast strains carrying *LoNAC5* exhibited significantly enhanced tolerance to both drought and salt stress. These findings strongly suggest that *LoNAC5* may act as a positive regulator under drought and salt stress, providing a certain theoretical basis for subsequent research. The role of *LoNAC5* in the drought and salt resistance of larch is worthy of further investigation.

ABA coordinates the plant stress response and regulates complex metabolic and physiological mechanisms that are vital for survival in a dynamically changing environment [67]. MeJA is a member of the jasmonic acid family, playing an important role in plant stress response [68]. Jasmonate- and ABA-mediated signaling pathways play crucial roles in activating plants’ defense responses against both biotic and abiotic stresses [69]. *ANAC019/055/072* may play a dual role in regulating ABA response and jasmonate response [70]. Additionally, *ANAC019/055/072* are crucial in the signal transduction of ABA-induced leaf senescence [63]. In this research, it was revealed that the promoter of *LoNAC5* harbors multiple elements associated with plant stress resistance, along with MeJA-responsive elements and ABA-responsive elements. The results of RT-qPCR indicate that the expression of *LoNAC5* is not only induced by drought and salt, but also significantly altered under the treatments of ABA and MeJA. In the larch seedlings treated with MeJA, the expression of *LoNAC5* was down-regulated. In contrast, after treatment with ABA, the expression of *LoNAC5* was up-regulated. The regulation of *LoNAC5* by MeJA and ABA may trigger a cascade of events, potentially activating downstream genes involved in osmolyte synthesis, ion homeostasis, and antioxidant defense, which are crucial for plant adaptation to abiotic stresses. Moreover, *LoNAC5* expression is robust in and specific to larch needles, indicating its potential functional significance in this specific tissue. Collectively, the evidence strongly suggests that *LoNAC5* may also exert a dual regulatory function in modulating both MeJA and ABA responses. It is highly likely to participate in the regulation of plant tolerance to abiotic stresses via the Jasmonate- and ABA-mediated signaling. However, further in-depth investigations are warranted to fully elucidate the role of *LoNAC5* in plant resistance to drought and salt stresses.

Transcription factors can bind to specific cis-acting elements on gene promoters to regulate gene expression [71]. CATGTG is an MYC-like sequence that plays a crucial role in the dehydration- and salt-induced expression of the *Arabidopsis ERD1* [72]. This sequence has important functions in many promoters that respond to stresses such as drought, salt, and abscisic acid [73]. Previous analyses of stress-inducible genes in *Arabidopsis* have demonstrated that, under diverse stress conditions, predominantly drought and salinity, the sequence CATGTG is pivotal in regulating gene expression [74]. Researchers have previously used the yeast one-hybrid system to isolate *ANAC019/055/072*, which can bind to the CATGTG motif in the promoter region of erd1 [6]. This study found that *LoNAC5* can also specifically bind to the CATGTG motif in yeast. It is speculated that *LoNAC5* binds to the CATGTG motif to regulate downstream genes, thereby acting as a positive regulator of drought and salt stress tolerance. Plant transcription factors are classified according to their characteristic DNA-binding domains. Given the functional significance of *LoNAC5*’s binding to the CATGTG motif, we further explored its potential interactions with other *LoNACs*. Studies have shown that even genes within the same family can be divided into different subgroups based on sequence information. Genes within the same subgroup have more similar gene structures and often occupy the same position in the regulatory network and play similar roles [17,75]. This study demonstrated that *LoNAC5* is capable of forming homodimers. Given the higher structural conservation among *LoNAC5* and *LoNAC7*/*14*/*15*, which belong to the same subgroup, it is hypothesized that *LoNAC7*/*14*/*15* are more likely to form heterodimers with *LoNAC5* than homodimers. These heterodimers may collaborate to participate in the plant stress-resistance process.

In summary, several critical scientific questions demand meticulous attention and in-depth investigation. Firstly, the molecular mechanisms by which *LoNAC5* modulates plant responses to abiotic stresses via Jasmonate- and ABA-mediated signaling cascades should be elucidated. Secondly, the potential formation of heterodimers between *LoNAC5* and *LoNAC7/14/15* (members of the stress-responsive NAC subgroup) and their subsequent roles in regulating the expression of downstream target genes should be explored. Addressing these scientific queries will not only facilitate the construction of a comprehensive molecular regulatory network in larch but also enable the identification and characterization of functionally significant genes. These efforts will ultimately provide invaluable genetic resources and a robust theoretical framework for the genetic improvement of larch, thereby enhancing its adaptability to adverse environmental conditions and promoting sustainable forestry practices.

## 4. Materials and Methods

### 4.1. Isolation of LoNAC5 and LoNAC5 Promoter

Based on the transcriptome data and the *L*. *kaempferi* genome from NCBI (taxid: 54,800, GenBank assembly GCA_013171265.2 Nucleotide BLAST), cloning primers for *LoNAC5* and its promoter were designed. *LoNAC5* forward primers were (5′-3′): ATGGGGAGGCAGAATGCA; reverse primers were (5′-3′): CTAATAAGAAGACCTTTGTAAATACCC. The *LoNAC5* promoter forward primers (5′-3′) were: CCCGGGATATGTGGTGGGTGGGTGG; reverse primers (5′-3′) were: CCATGGGTTGTTAAAATTGCTATCGATTTAAC. cDNA and gDNA from *L. olgensis* were used as templates for PCR amplification. After purifying the target DNA bands (E.Z.N.A.^®^ Gel Extraction Kit, Omega Bio-Tek, Norcross, Georgia), the sample was dispatched to Sangon Biotech Co., Ltd. (Shanghai, China) for Sanger sequencing to acquire the sequence information of *LoNAC5* and its promoter.

### 4.2. Web Tools and Software Used for Bioinformatics Analysis

A total of 15 NAC proteins of *L*. *olgensis*, including LoNAC5 (GenBank accession: MW206687–MW206691 and OQ941818–OQ941827), which were retrieved from NCBI, along with the NAC proteins of *Arabidopsis thaliana* obtained from the database (https://www.arabidopsis.org/browse/genefamily/NAC.jsp (accessed on 8 June 2024)), were employed to construct a neighbor-joining phylogenetic tree using the MEGA 5.0 software [76]. Subgroup classification was conducted by referring to the existing research findings, and the phylogenetic tree was refined and formatted using Evolview (https://www.evolgenius.info/evolview/ (accessed on 10 June 2024)). The NCBI CD search was utilized to predict and analyze the domains of these proteins, while MEME (https://meme-suite.org/meme/tools/meme (accessed on 10 June 2024)) was applied to analyze their motifs. GOR4 (https://npsa-prabi.ibcp.fr/cgi-bin/npsa_automat.pl?page=npsa_gor4.html (accessed on 10 November 2024)) and SwissModel (https://www.swissmodel.expasy.org/ (accessed on 10 November 2024)) were, respectively, used to predict the secondary structure and the tertiary structure of the LoNAC5 protein. The prediction of signal peptides and transmembrane domains was accomplished by SignalP 6.0 (https://services.healthtech.dtu.dk/services/SignalP-6.0/ (accessed on 10 November 2024)) and DeepTMHMM (https://dtu.biolib.com/DeepTMHMM (accessed on 10 November 2024)), respectively. Furthermore, the PlantCARE website (http://bioinformatics.psb.ugent.be/webtools/plantcare/html/ (accessed on 13 February 2024)) was exploited to analyze the cis-acting elements existing in the promoter region.

### 4.3. Analysis of LoNAC5 Promoter Activity

The *LoNAC5* promoter was inserted between the XmaI and NcoI cleavage sites of the VB191103-1905rcy plant expression vector, resulting in the construction of a recombinant plasmid, designated as *LoNAC5*pro::GUS. Subsequently, *Agrobacterium* GV3101 cells were transformed with either the recombinant plasmid or the empty vector plasmid. The transformed *Agrobacterium* cells were then cultured in LB medium supplemented with 50 mg/L Kanamycin (Kan) and Rifampicin (Rif) at 200 rpm and 28 °C. The cultivation was continued until OD_600_ ≈ 0.8, at which point the cells were ready for subsequent experiments. Using one-week-old tobacco seedlings as experimental materials, they were immersed in *Agrobacterium* with *LoNAC5*pro::GUS or 35S::GUS resuspended in 1/2 MS medium (OD₆₀₀ = 0.5–0.6). After shaking for 3–4 h at 120 rpm and 25 °C, the seedlings were placed on 1/2 MS medium and incubated in the dark at 25 °C for 2 days. Then, they were put into the prepared GUS staining solution and incubated in the dark at 37 °C for 12 h. Finally, the stained tobacco seedlings were placed in 95% ethanol for decolorization.

### 4.4. Subcellular Localization of LoNAC5

The *LoNAC5* was inserted between the BamHI and HindIII restriction sites of the plant expression vector VB191104-GFP to obtain the recombinant plasmid (VB191104-*LoNAC5*-GFP). *Agrobacterium* harboring the recombinant plasmid and empty vector plasmid were cultured in LB medium containing 50 mg/L of both Kan and Rif at 28 °C with a shaking speed of 200 rpm until OD_600_ ≈ 0.8. After that, the *Agrobacterium* was resuspended in the prepared 1/2 MS solution to OD_600_ = 0.5–0.6. The infection solution was then loaded into a syringe. Using the leaves of *Nicotiana benthamiana* seedlings that had grown for about one month as experimental materials, the infection solution was injected into the tobacco leaves through the lower epidermis. The injected tobacco plants were then incubated in the dark at 25 °C for 2–3 days. Slices were made from the injected tobacco leaves, and the GFP signals were detected using a laser-confocal fluorescence microscope.

### 4.5. Research on Mechanism of LoNAC5

The full-length and fragments of *LoNAC5* were, respectively, inserted between the BamHI and EcoRI restriction sites of pGBKT7. After the recombinant plasmids that were confirmed to be correct by sequencing were transformed into Y2HGold yeast strain, they were spread on SD/-Trp medium and incubated upside-down at 30 °C for 1–2 days. Single colonies were picked and suspended in 10 μL of physiological saline. After the bacterial suspension was verified to be correct, the remaining bacterial suspension was spotted on SD/-Trp/-His/-Ade/X-α-Gal screening medium and incubated upside-down at 30 °C for 2–3 days to observe the growth of the yeast. After inserting *LoNAC5* into the SmaI site of pGADT7 to obtain the recombinant plasmid, it was co-transformed with the pGBKT7-*LoNAC5* fragment plasmid without self-activation activity into the Y2HGold yeast strain. Then, the growth states of the yeast were observed after spreading the yeast bacterial suspension on DDO and QDO/X media, respectively. The classic NAC recognition sequence (NACRS)-CATGTG was repeated three times and inserted between the EcoRI and SacI restriction sites of the pHIS2. After obtaining the recombinant plasmid, it was co-transformed with pGADT7-*LoNAC5* into Y187 yeast strain. The yeast suspension was spread on DDO and TDO media, respectively. After incubating the plates upside-down at 30 °C for 3–5 days, the growth of the yeast was observed.

### 4.6. Functional Characterization of LoNAC5

The plasmids of pYES2-NTB-*LoNAC5* (experimental group) and pYES2-NTB (control group) were, respectively, transformed into the INVSC1 yeast strain. Positive clones were picked and resuspended in 2 mL of sterile water. After dilution (10^0^, 10^−1^, 10^−2^), the suspensions were spread on the following plates: SG-U + 0 mM PEG 3350, SG-U + 30 mM PEG 3350, SG-U + 60 mM PEG 3350, SG-U + 90 mM PEG 3350, SG-U + 120 mM PEG 3350, SG-U + 135 mM PEG 3350; SG-U + 0 M NaCl, SG-U + 0.5 M NaCl, SG-U + 1.0 M NaCl, SG-U + 1.3 M NaCl, SG-U + 1.5 M NaCl, and SG-U + 2.0 M NaCl (Merck KGaA, Darmstadt, Germany). The plates were then placed in an incubator at 30 °C. After 7 days, the plates were observed and photographed. The lithium acetate method was applied to the transformation of all the yeast strains mentioned above [27,77].

### 4.7. Analysis of LoNAC5 Expression in L. olgensis

*L. olgensis* at different growth stages (2-month-old, 4-month-old, and 6-month-old) were used as experimental materials. The roots, stems, and needles of the plants were separated and sampled. Separately, different abiotic stress treatments (28% *w/v* PEG 6000, 250 mmol·L^−1^ NaCl, 50 mmol·L^−1^ NaHCO_3_, from Merck KGaA) and hormone treatments (50 mg·L^−1^ IAA, GA_3_, MeJA, ABA and 2,4-D, from PhytoTech Labs, Lenexa, KS, USA) were applied to 2-month-old seedlings. Each treatment was applied once at 0 h, 48 h, 72 h, and 84 h, with water as the control. Sampling was carried out after 96 h of treatment [53]. Total RNA was extracted from the samples using the Universal Plant Total RNA Extraction Kit (BIOTEKE, Beijing, China) according to the manufacturer’s protocol. Subsequently, the extracted total RNA was reverse transcribed into cDNA using the ReverseScript RT reagent Kit (TaKaRa, Shiga, Japan). The synthesized cDNA was then employed as the template for subsequent experiments.

The primers for RT-qPCR were designed with Primer Premier. The forward primer was (5′-3′): CTGATGTCAATAGGACTGCAAAG, and the reverse primer was (5′-3′): ATTCCTGGCACCTTTTCATC. These primers were meticulously screened via gel electrophoresis detection. The *LoTubulin* primers, as reported in reference [52,53], were selected as the internal control. Using different cDNA templates of *L. olgensis*, samples were loaded following the 20 μL reaction system specified in the TransStart^®^ Top Green qPCR SuperMix Top (TransGen Biotech, Beijing, China). Amplification was then performed using an ABI 7500 real-time PCR instrument. In RT-qPCR, the results were analyzed using the 2^−ΔΔCt^ method [78].

## 5. Conclusions

In this study, the NAC family transcription factor *LoNAC5* was isolated from *L*. *olgensis*, which comprises three exons and two introns. The length of the CDS region is 1164 bp, encoding 387 amino acids. In the LoNAC5 protein there is a NAM domain located at positions 14aa to 139aa of the N-terminus, and a transcriptional activation domain at positions 324aa to 364aa of the C-terminus. The subcellular localization of *LoNAC5* is in the cell nucleus. Overall, *LoNAC5* is a typical transcription factor of the NAC family. Since *LoNAC5* belongs to the AtNAC3 subgroup, it can be classified as a stress-responsive NAC. Through a series of well-designed experiments, we have provided solid evidence that *LoNAC5* is more strongly expressed in plant leaves, can participate in the responses to ABA and MeJA, and plays a positive regulatory role in the processes of drought and salt tolerance. The yeast experiment results showed that *LoNAC5* can form a homodimer and can specifically bind to the CATGTG motif. These findings lay a solid foundation for subsequent exploration of the molecular mechanisms underlying *LoNAC5*-mediated regulatory pathways.

## Data Availability

Data are contained within the article and can be made available on request.
